# Large language model-based uncertainty-adjusted label extraction for artificial intelligence model development in upper extremity radiography

**DOI:** 10.1007/s00330-025-12102-1

**Published:** 2025-11-14

**Authors:** Hanna Kreutzer, Anne-Sophie Caselitz, Thomas Dratsch, Daniel Pinto dos Santos, Christiane Kuhl, Daniel Truhn, Sven Nebelung

**Affiliations:** 1 Department of Diagnostic and Interventional Radiology, University Hospital Aachen, Aachen, Germany; 2 Lab for Artificial Intelligence in Medicine, Department of Diagnostic and Interventional Radiology, University Hospital Aachen, Aachen, Germany; 3 Institute for Diagnostic and Interventional Radiology, Faculty of Medicine and University Hospital Cologne, University of Cologne, Cologne, Germany; 4 Department of Diagnostic and Interventional Radiology, University Medical Center Mainz, Mainz, Germany

**Keywords:** Radiography, Large language models, Artificial intelligence, Upper extremity

## Abstract

**Objectives:**

To evaluate GPT-4o’s zero-shot ability to extract structured diagnostic labels (with uncertainty) from free-text radiology reports and to test how these labels affect multi-label image classification of musculoskeletal radiographs.

**Materials and methods:**

This retrospective study included radiography series of the clavicle (*n* = 1170), elbow (*n* = 3755), and thumb (*n* = 1978). After anonymization, GPT-4o filled out structured templates by indicating imaging findings as present (“true”), absent (“false”), or “uncertain.” To assess the impact of label uncertainty, “uncertain” labels of the training and validation sets were automatically reassigned to “true” (inclusive) or “false” (exclusive). Label-image pairs were used for multi-label classification using the ResNet50 architecture. Label extraction accuracy was manually verified on internal (clavicle: *n* = 233, elbow: *n* = 745, thumb: *n* = 393) and external test sets (*n* = 300 for each). Performance was assessed using macro-averaged receiver operating characteristic (ROC) area under the curve (AUC), precision, recall curves, sensitivity, specificity, and accuracy. AUCs were compared with the DeLong test.

**Results:**

Automatic extraction was correct in 98.6% (60,618 of 61,488) of labels in the test sets. Across anatomic regions, label-based model training yielded competitive performance measured by macro-averaged AUC values for inclusive (e.g., elbow: AUC = 0.80 (range, 0.62–0.87)) and exclusive models (elbow: AUC = 0.80 (range, 0.61–0.88)). Models generalized well on external datasets (elbow (inclusive): AUC = 0.79 (range, 0.61–0.87); elbow (exclusive): AUC = 0.79 (range, 0.63–0.89)). No significant differences were observed across labeling strategies or datasets (*p *≥ 0.15).

**Conclusion:**

GPT-4o extracted labels from radiologic reports to train competitive multi-label classification models with high accuracy. Detected uncertainty in the radiologic reports did not influence the performance of these models.

**Key Points:**

***Question*** *Can GPT-4o automatically extract high-accuracy, uncertainty-aware diagnostic labels from routine radiologic reports of the clavicle, elbow, and thumb for use in training multi-label image classifiers?*

***Findings*** *GPT-4o extracted labels with > 98% accuracy, and multi-label classifiers for clavicle, elbow, and thumb radiographs performed consistently regardless of how uncertainty was handled.*

***Clinical relevance*** *Automated GPT-4o-based labeling of routine clavicle, elbow, and thumb radiologic reports enables the rapid conversion of radiologic reports into structured multi-label training datasets, supporting scalable development of dedicated image classification models.*

**Graphical Abstract:**

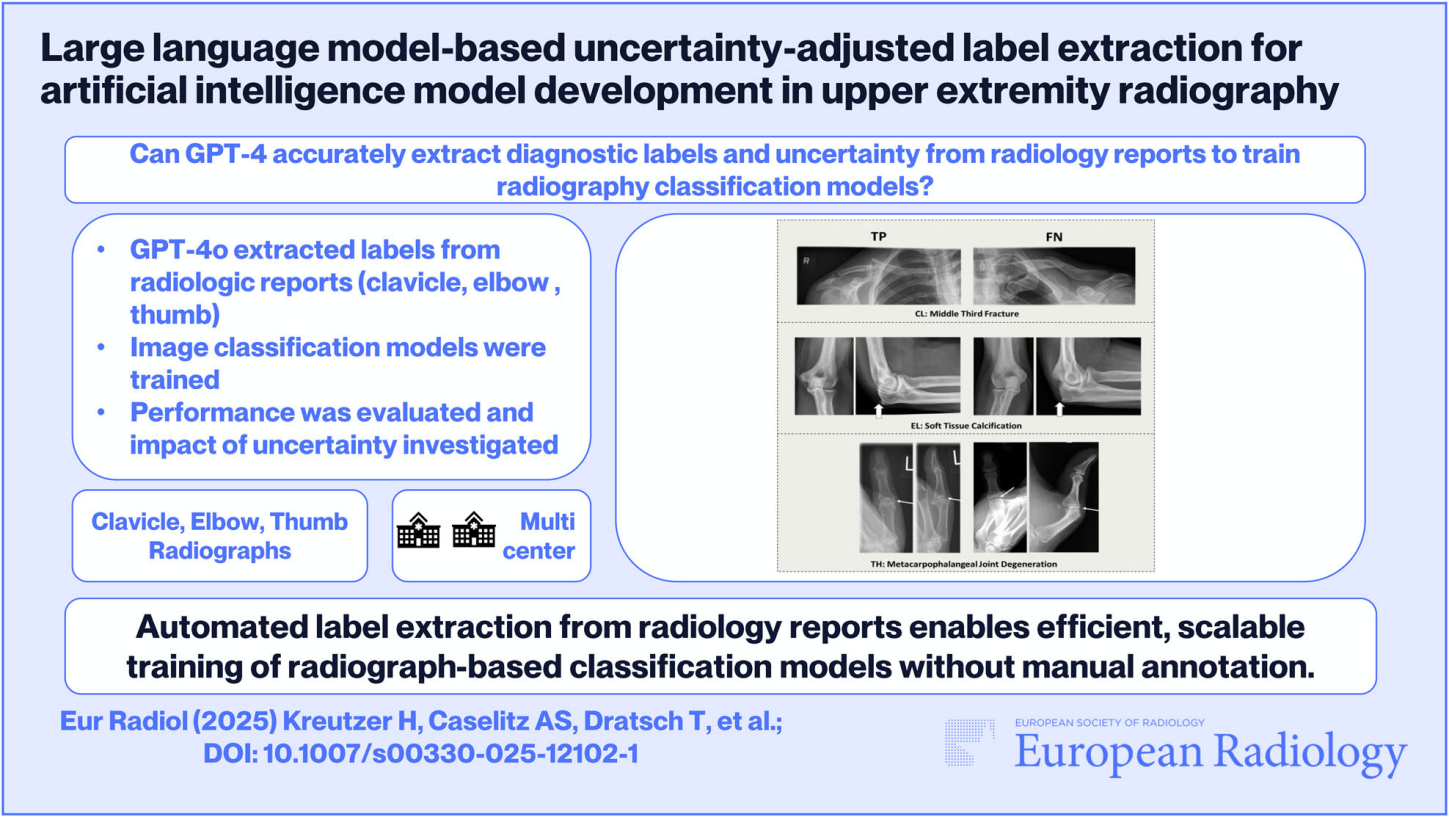

## Introduction

An estimated 3.6 billion imaging procedures are conducted globally each year, according to the World Health Organization [1]. Despite this abundance, AI model development is challenged by data scarcity [2]. Increasing the availability of imaging study-report pairs would enhance data quantity and variability for AI model training and help to address performance issues, such as weak generalizability [3]. Traditionally, such datasets are created through manual annotation, an approach that is labor-intensive, costly, and, if performed by non-experts, inconsistent in quality [4]. Automated label extraction from radiologic reports has also been attempted using rule-based or conventional machine-learning natural language processing (NLP) methods [5]. While promising [6–9], these require additional training and often struggle with terminological complexity. Mislabeling rates of up to 10% have been reported [10], potentially degrading model performance.

Large language models (LLMs) offer a more adaptable alternative for processing radiologic reports, with the ability to interpret complex linguistic patterns and transform free text into structured templates [11, 12]. For instance, Al Mohamad et al used an open-source LLM (Mixtral 8 × 7b) to extract binary fracture labels from ankle radiographs and train a standard convolutional neural network (CNN) [13]. However, their study addressed a single label in one anatomic region and did not account for label uncertainty, i.e., phrases such as “likely,” “suggestive,” or “indeterminate”, that are common in radiology reports [14] and reflect inherent diagnostic ambiguity. Ignoring uncertainty risks introducing noise into datasets, potentially impairing downstream AI performance.

To our knowledge, no prior study has explored automated, uncertainty-aware label extraction using LLMs across multiple upper-extremity regions for training multi-label classification models. While fracture-detection AI models in the upper extremity have been reviewed [15], comprehensive models addressing both common and less frequent conditions of the clavicle, elbow, and thumb remain understudied.

Our objective was to investigate whether LLMs can be used for automated label extraction across multiple anatomic regions of the upper extremity to train multi-label classification models and whether label uncertainty impacts model performance. We hypothesized that (i) LLMs can accurately extract labels from radiologic reports while detecting uncertainty, (ii) label uncertainty does not affect model performance, and (iii) extracted labels enable efficient training of multi-label classification models.

## Methods

### Study design

This study was designed as a two-center retrospective analysis utilizing radiography series and original radiologic reports of the elbow, thumb, and clavicle. The internal dataset from our university hospital (Department of Diagnostic and Interventional Radiology, University Hospital Aachen) was sourced from our local Picture Archiving and Communication System (isite, Philips), spanning 2010 to 2024. The study was conducted in accordance with local data protection laws, following approval from the local ethical committee (Ethical Committee, Medical Faculty, RWTH Aachen University, EK24-174), with a waiver for individual informed consent. External test sets were collected from the University Hospital Cologne (Institute of Diagnostic and Interventional Radiology) with ethical approval (24-1348-retro) and a waiver for informed consent. Radiographs at the internal site were acquired using five computed-radiography units: Philips DigitalDiagnost VR, Philips DigitalDiagnost 4 High Performance, Siemens Ysio Max, and two Philips DigitalDiagnost C90 systems. Radiographs at the external site were acquired using a Philips DigitalDiagnost system. All units employed flat-panel detectors, automatic exposure control, and the site’s standard radiography protocols. Routine upper-extremity exposure settings were 50–60 kVp and 2–5 mAs for the elbow and thumb, and 60–70 kVp, 8–18 mAs for the clavicle, using a 100 cm source-to-image distance.

### Patient selection

**Fig. 1:**
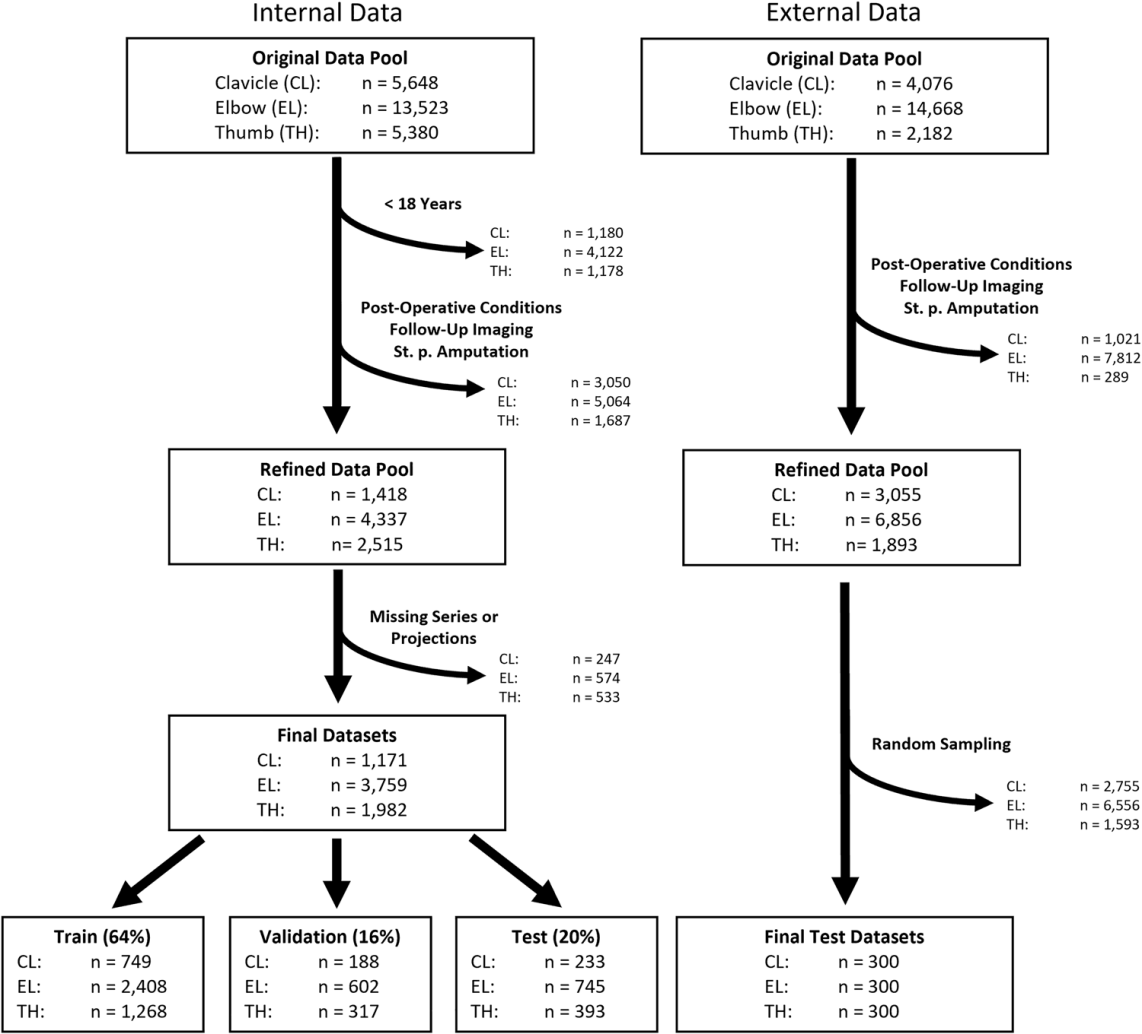
Data Curation and Preparation. Left: internal dataset (University Hospital Aachen, 2010–2024); right: external dataset (University Hospital Cologne, 2010–2022). Identical exclusion criteria were applied to both sources: patients < 18 years, post-operative imaging, follow-up examinations, and studies after amputation. Pediatric cases had already been removed by the Cologne site (note beneath the age-exclusion box). After exclusions, the Cologne pool underwent region-stratified random sampling of 300 studies each of the clavicle (CL), elbow (EL), and thumb (TH). The internal data were split into training (64%), validation (16%), and internal test (20%) subsets. The external data was served only for final testing. All final datasets comprise anteroposterior projections of the clavicle and both anteroposterior and lateral projections of the elbow and thumb “St. p., status post”

Patient selection is visualized in Fig. 1. The final internal datasets were split into training (64%), validation (16%), and test sets (20%) while ensuring balanced label distribution across the sets. Because this retrospective study used all examinations that met the inclusion criteria, no a priori sample-size calculation was performed. For the external data, the collaborating hospital had already removed studies of patients < 18 years and examinations with missing projections. Therefore, only post-operative, follow-up, and amputation cases had to be excluded locally. An automated Python script (v 3.12.3) then drew a random sample of 300 patients per anatomic region for the final external test sets.

### Overall approach

**Fig. 2:**
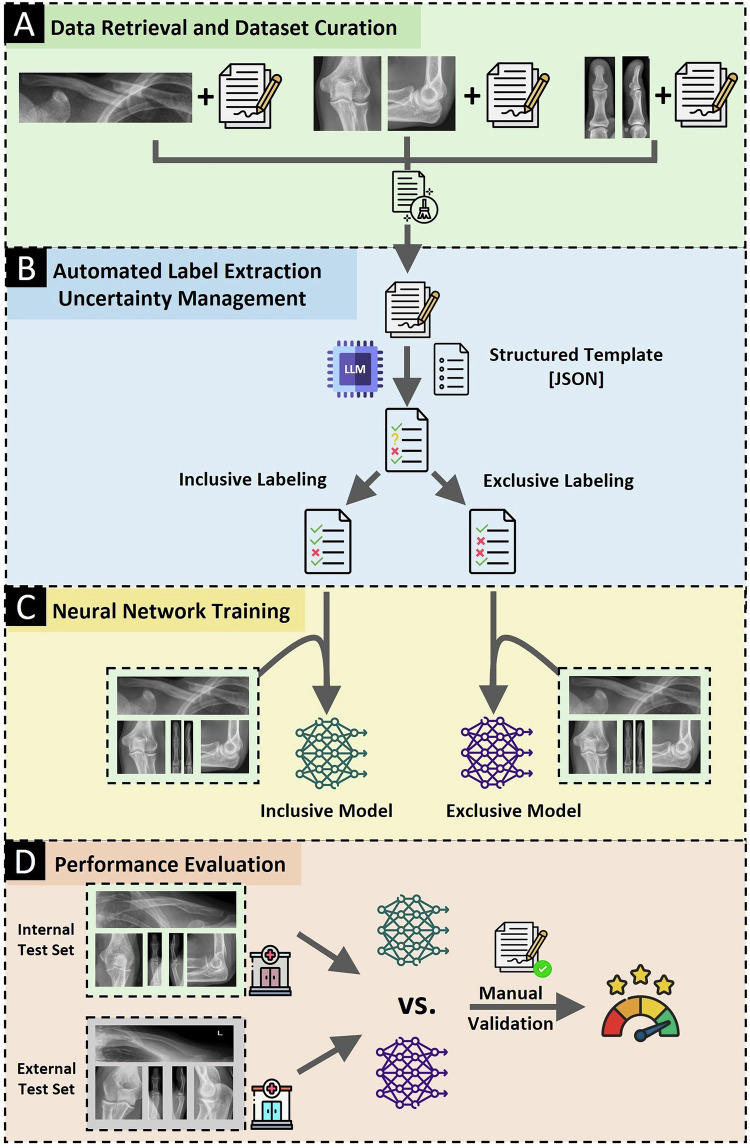
Study Workflow. **A** Radiography series of the clavicle, elbow, and thumb and corresponding radiologic reports were collected and curated. **B** The LLM filled out a region-specific structured template containing relevant conditions based on the radiologic reports. Individual labels were either “true,” false,” or “uncertain.” The template was machine-readable and available in the JavaScript Object Notation (JSON) format. For subsequent model training, “uncertain” labels of the training/validation sets were automatically converted into “true” (inclusive labeling) or “false” (exclusive labeling) using Python. **C** The JSON files were paired with the radiography series to train the image classification models as inclusive and exclusive versions using the respective labels. **D** Both models were tested on internal and external test datasets containing manually corrected labels (ground truth)

The general procedure is illustrated in Fig. 2. OpenAI’s GPT-4o was used to extract structured labels from free-text radiologic reports, which were used to train dedicated CNNs for classification. Python was used for data processing and implementation of the models.

#### Automatic label extraction

Before label extraction, reports were automatically anonymized by removing personal identifiers such as patient names, physician details, dates, and patient IDs. Successful anonymization was manually verified. GPT-4o was used in a zero-shot manner, i.e., operated without prior task-specific fine-tuning on label extraction (Supplementary Text 1). The tool was instructed to fill out predefined JavaScript Object Notation (JSON) templates for each anatomic region that contained common conditions like fractures and less common findings like sclerotic lesions and soft-tissue masses (Supplementary Text 2). The templates were designed by a senior MSK radiologist (S.N., with 12 years of clinical experience).

For each label (pathology), the tool was instructed to choose from three options: “true”, “false”, or “uncertain.” “True” indicates that the imaging finding of interest is present (pathology present), “false” indicates that the finding is absent (pathology absent), and “uncertain” indicates that the report contains expressions of diagnostic uncertainty. Consequently, labels were marked as “uncertain” when reports contained phrases indicating doubt or hedging terms. The uncertainty terms were based on dedicated literature [14, 16] and included expressions such as “possibly” and “suspected.” These examples were provided along with their German translations since the reports were in German.

#### Manual quality check

Following the automated label extraction, a medical student (A.C.) was trained and supervised by the senior MSK radiologist (S.N.) to manually verify and correct the labels for the internal and external test sets. The verification process involved comparing the extracted labels with the original reports. “Uncertain” labels were not allowed in the test, and thus, follow-up imaging studies were reviewed to definitively categorize each label as “true” (pathology present) or “false” (pathology absent).

#### Model training

Model training was implemented using PyTorch (version 2.4.0) [17]. For each anatomic region, we trained two model variants that differed only in how uncertain labels were handled during training and validation. In the inclusive model, all labels originally marked as “uncertain” during label extraction (e.g., a report phrase such as “possible fracture”) were converted to “true” (finding present), thereby treating uncertain findings as positive cases. In the exclusive model, all “uncertain” labels were converted to “false” (finding absent), thereby treating uncertain findings as negative cases. Overall, the only difference between the two models was the handling of these “uncertain” labels in the training and validation datasets. All other parameters and architectures remained identical. Definitive “true” and “false” labels were left unchanged in the training and validation sets, and all test sets contained only definitive labels. Each model was based on a modified ResNet50 architecture [18]: The original 1000-class output layer was replaced with a new fully connected layer to output a vector of logits corresponding to each condition’s binary classification, i.e., presence or absence. These logits were then passed through a sigmoid activation function to convert them into probabilities. For elbow and thumb datasets, which included both anteroposterior and lateral projections, each image was processed through separate ResNet50 networks with identity layers as final layers; their extracted feature vectors were concatenated before being fed into a final fully connected layer.

We used the AdamW optimizer initialized for training with a learning rate of 0.001. A step-based scheduler (StepLR) was employed to reduce the learning rate by 0.1 every seventh epoch.

Preprocessing included resizing the images to 512 × 512 pixels and augmentation with random flips, rotation, and color jitter.

The optimal decision threshold for the test set was determined on the validation set using the Youden index method [19]. A summary of all training details can be found in Supplementary Table S1.

### Statistical analysis

Statistical analyses were performed using R software (version 4.3.1; R Project for Statistical Computing) and Python. Label extraction performance was assessed using two metrics: (1) label-level accuracy, i.e., the proportion of labels correctly extracted across all reports, and (2) report-level accuracy, i.e., the proportion of reports where all labels were correctly extracted. Model performance metrics included accuracy, sensitivity, specificity, Area Under the Receiver Operating Characteristic Curve (AUC) and precision-recall curves. Macro-averaged AUC values, i.e., the unweighted AUC averages computed for each label, were calculated across all labels with at least 10 positive cases in the internal and external test sets. This threshold was selected to strike a sensible balance between sufficient sample sizes for reliable AUC estimates and comprehensive multi-label evaluation. The DeLong test [20] was used to compare AUC values between inclusive and exclusive models and between internal and external test sets, using the R pROC package [21]. Confidence intervals were determined by 1000 bootstrap replicates, and the Benjamini–Hochberg correction [22] was applied to account for multiple comparisons against the significance threshold of *p* < 0.05.

## Results

Patient demographics are detailed in Table 1. Counts of conditions as a function of dataset, split, and model type are provided in Supplementary Tables S2–S4.

**Table 1:** Patient demographics as a function of dataset (internal, external), split, and anatomic region

Dataset	Anatomic region	Split	*N*	Age (years)	Women
Internal		Train	749	46.4 ± 20.7	244 (32.6)
	Clavicle	Validation	188	45.6 ± 20.8	47 (25.0)
		Test	233	45.4 ± 19.8	80 (34.3)
		Train	2408	48.4 ± 20.8	1031 (42.9)
	Elbow	Validation	602	49.0 ± 20.3	271 (45.2)
		Test	745	48.5 ± 20.9	305 (40.9)
		Train	1268	41.6 ± 17.4	482 (38.0)
	Thumb	Validation	317	41.1 ± 17.6	132 (41.6)
		Test	393	41.9 ± 18.2	158 (40.2)
External	Clavicle	Test	300	47.3 ± 18.5	113 (37.7)
	Elbow	Test	300	45.5 ± 17.8	127 (42.3)
	Thumb	Test	300	41.7 ± 16.0	132 (44.0)

Patient age is presented as mean ± standard deviation. Patient sex is presented as a count (%)

The label extraction process functioned as intended, and the tool successfully filled out the templates using the specified JSON formats. For the internal data, clavicle reports achieved 98.8% label-level accuracy (5988/6058) and 78.5% report-level accuracy (183/233 reports with 26 labels each). Elbow reports reached 98.6% (21,294/21,605) and 74.4% (554/745, 29 labels per report), whereas thumb reports achieved 99.0% (9731/9825) and 85.5% (336/393, 25 labels per report).

In the external test set of 300 radiologic reports per joint, label extraction performance was similarly strong: For the clavicle, label-level accuracy was 98.6% (7687/7800) and report-level accuracy was 72.7% (218/300). For the elbow, the accuracy rates were 98.4% (8564/8700) and 71.3% (214/300), and for the thumb, they were 98.1% (7354/7500) and 73.7% (221/300).

Detecting uncertain labels posed a challenge. For the clavicle, manual identification found uncertain labels in 3.9% (9/233) of reports, but only two (0.9%) were automatically detected. For the elbow, uncertain labels were present in 10.5% (78/745) of reports, with the tool identifying 48 (6.4%). For the thumb, uncertain labels were present in 9.7% (38/393), with the tool identifying 21 (5.3%). A similar trend was observed for the external test set: Uncertainty was present in 5.3% (16/300; clavicle), 16.3% (49/300; elbow), and 16.0% (48/300; thumb), respectively, of which the extraction pipeline detected 10 (3.3%), 26 (8.7%), and 40 (13.3%).

The training and validation sets contained few such labels—42 for clavicle, 492 for elbow, and 231 for thumb (Supplementary Table S5).

**Fig. 3:**
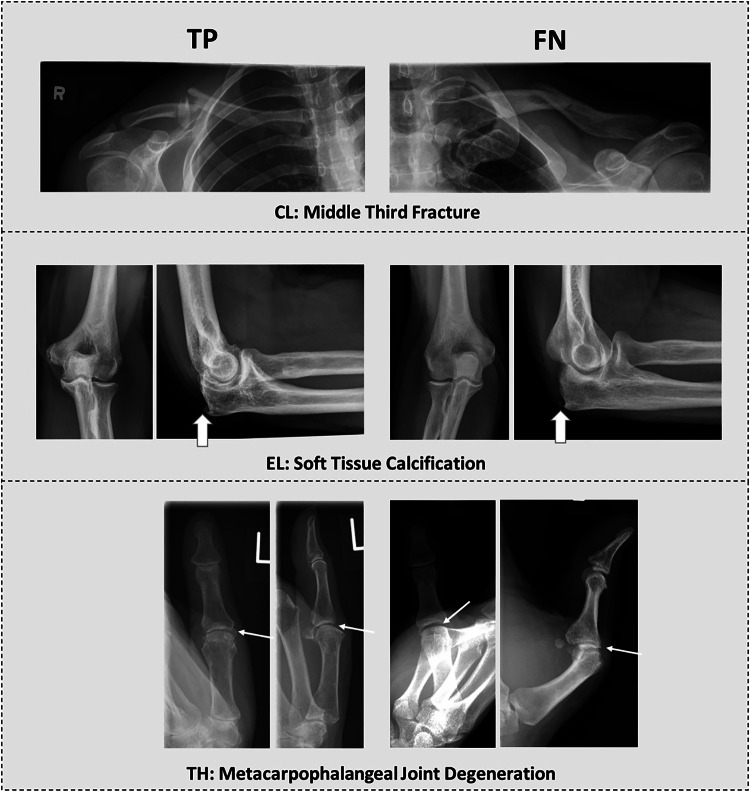
Representative radiography series illustrating model performance across different labels and anatomic regions. Shown are true positives (TP; left) and false negatives (FN; right) for the label indicated underneath each radiography series consisting of anteroposterior (left) and lateral projections (right). For the clavicle (CL), the model correctly identified a middle-third fracture in one patient (TP) and misclassified it as a medial third fracture in another patient (FN). For the elbow (EL), a soft-tissue calcification at the triceps tendon insertion was accurately detected in one patient (TP) and missed in another (FN), likely because of its fainter and more subtle appearance. For the thumb (TH), metacarpophalangeal joint degeneration was correctly identified in one patient (TP) and missed in another patient (FN), likely because of a fixed Boutonnière-like deformity and consecutive superimposition of the metacarpus. Block arrows indicate calcifications, while arrows indicate signs of degeneration

The models achieved competitive performance across the anatomic regions, but per-label performance varied. A representative radiography series illustrating model performance across different labels and anatomic regions is shown in Fig. 3.

Tables 2–4 detail AUC values (for labels with *n* ≥ 10) and *p*-values of the DeLong test, while additional performance metrics, i.e., accuracy, sensitivity, and specificity as well as ROC and precision-recall curves, can be found in the supplement (Supplementary Tables S6–S11; Supplementary Figs. S1–S3). In brief, these precision recall curves reflected the impact of class imbalance, with rare labels (e.g., “Ossicles” (elbow and thumb)) showing steep early precision drop-offs despite moderate AUC values, whereas high-prevalence labels (e.g., “Fracture (All Locations)” (elbow and thumb)) maintained high precision across a broader recall range. Across all regions, both model variants tended to demonstrate high specificity for rare findings but reduced sensitivity, particularly for subtle or soft tissue-related abnormalities.

**Table 2:** Performance of the image classification models trained with automatically extracted labels for radiographs of the clavicle

Label	Dataset	*N*	Inclusive model	Exclusive model	*p*-value (**)
	Internal	121	0.95 (0.91, 0.97)	0.94 (0.90, 0.96)	0.81
Fracture (all locations)	External	147	0.91 (0.88, 0.94)	0.91 (0.88, 0.95)	0.95
	*p*-value (*)		0.73	0.73	
	Internal	98	0.91 (0.87, 0.94)	0.90 (0.85, 0.94)	0.91
Displacement	External	123	0.93 (0.90, 0.95)	0.92 (0.88, 0.95)	0.81
	*p*-value (*)		0.73	0.89	
	Internal	43	0.89 (0.84, 0.93)	0.89 (0.84, 0.93)	0.95
Comminuted or fragmented fracture	External	59	0.91 (0.87, 0.94)	0.91 (0.87, 0.94)	0.95
	*p*-value (*)		0.76	0.73	
	Internal	66	0.84 (0.78, 0.89)	0.86 (0.80, 0.91)	0.81
Middle-third fracture	External	76	0.90 (0.86, 0.94)	0.88 (0.84, 0.92)	0.64
	*p*-value (*)		0.73	0.73	
	Internal	45	0.83 (0.75, 0.89)	0.83 (0.76, 0.90)	0.95
Lateral third fracture	External	67	0.81 (0.75, 0.87)	0.83 (0.77, 0.88)	0.81
	*p*-value (*)		0.91	0.91	
	Internal	18	0.78 (0.65, 0.89)	0.77 (0.64, 0.87)	0.91
Acromioclavicular joint degeneration	External	28	0.74 (0.65, 0.82)	0.74 (0.65, 0.82)	0.95
	*p*-value (*)		0.91	0.91	
	Internal	19	0.78 (0.65, 0.90)	0.76 (0.64, 0.87)	0.81
Joint degeneration (all locations)	External	59	0.75 (0.68, 0.82)	0.76 (0.68, 0.83)	0.95
	*p*-value (*)		0.91	0.95	
	Internal	14	0.60 (0.46, 0.72)	0.63 (0.47, 0.77)	0.86
Acromioclavicular joint–joint space widened	External	34	0.53 (0.42, 0.64)	0.51 (0.41, 0.62)	0.91
	*p*-value (*)		0.81	0.81	
	Internal	12	0.59 (0.39, 0.75)	0.70 (0.55, 0.83)	0.49
Swelling or hematoma	External	34	0.47 (0.36, 0.58)	0.54 (0.42, 0.65)	0.46
	*p*-value (*)		0.81	0.64	

Area under the receiver operating characteristic curve (AUC) values are shown with 95% confidence intervals in parentheses for each label with at least 10 positive cases in both internal and external test sets. *N* refers to the number of positive cases in the corresponding test set, which was identical for the inclusive and exclusive models. *p*-values from the DeLong test compare (i) internal vs. external test sets for the same model (*, row-wise) and (ii) inclusive vs. exclusive models within the same test set (**, column-wise)

**Table 3:** Performance of the image classification models trained with automatically extracted labels for radiographs of the elbow

Label	Dataset	*N*	Inclusive model	Exclusive model	*p*-value (**)
	Internal	162	0.87 (0.83, 0.90)	0.88 (0.85, 0.91)	0.86
Fracture (all locations)	External	56	0.85 (0.79, 0.90)	0.85 (0.79, 0.90)	0.99
	*p*-value (*)		0.86	0.86	
	Internal	112	0.86 (0.82, 0.90)	0.85 (0.82, 0.89)	0.86
Radius fracture	External	44	0.84 (0.76, 0.91)	0.88 (0.81, 0.94)	0.41
	*p*-value (*)		0.92	0.86	
	Internal	102	0.86 (0.81, 0.90)	0.85 (0.81, 0.89)	0.86
Radial head fracture	External	42	0.87 (0.80, 0.93)	0.89 (0.84, 0.94)	0.73
	*p*-value (*)		0.89	0.73	
	Internal	57	0.82 (0.76, 0.87)	0.81 (0.76, 0.86)	0.86
Exostosis	External	40	0.86 (0.80, 0.91)	0.85 (0.78, 0.91)	0.86
	*p*-value (*)		0.86	0.86	
	Internal	52	0.81 (0.74, 0.86)	0.82 (0.75, 0.87)	0.86
Joint degeneration (all locations)	External	39	0.84 (0.77, 0.92)	0.84 (0.77, 0.91)	0.99
	*p*-value (*)		0.86	0.86	
	Internal	12	0.80 (0.66, 0.92)	0.85 (0.74, 0.94)	0.47
Sclerotic lesion	External	21	0.65 (0.53, 0.77)	0.63 (0.52, 0.75)	0.86
	*p*-value (*)		0.54	0.15	
	Internal	95	0.78 (0.72, 0.84)	0.78 (0.72, 0.84)	0.99
Fat pad sign	External	38	0.78 (0.68, 0.86)	0.78 (0.69, 0.86)	0.99
	*p*-value (*)		0.99	0.99	
	Internal	61	0.78 (0.71, 0.83)	0.76 (0.68, 0.83)	0.73
Soft-tissue calcifications	External	30	0.61 (0.49, 0.71)	0.63 (0.52, 0.73)	0.86
	*p*-value (*)		0.23	0.41	
	Internal	19	0.62 (0.48, 0.74)	0.61 (0.48, 0.72)	0.99
Ossicles	External	16	0.78 (0.66, 0.90)	0.75 (0.60, 0.87)	0.86
	*p*-value (*)		0.41	0.54	

Table organization as in Table 2

**Table 4:** Performance of the image classification models trained with automatically extracted labels for radiographs of the thumb

Label	Dataset	*N*	Inclusive model	Exclusive model	*p*-value (**)
	Internal	30	0.91 (0.85, 0.96)	0.90 (0.85, 0.95)	0.95
Carpometacarpal joint degeneration	External	30	0.90 (0.84, 0.95)	0.90 (0.85, 0.95)	0.96
	*p*-value (*)		0.95	0.96	
	Internal	52	0.89 (0.84, 0.93)	0.88 (0.81, 0.93)	0.69
Joint degeneration (all locations)	External	49	0.90 (0.85, 0.95)	0.91 (0.86, 0.95)	.073
	*p*-value (*)		0.95	0.71	
	Internal	21	0.88 (0.78, 0.95)	0.89 (0.79, 0.96)	0.73
Metacarpophalangeal joint degeneration	External	20	0.85 (0.75, 0.92)	0.85 (0.77, 0.93)	1
	*p*-value (*)		0.72	0.54	
	Internal	14	0.85 (0.69, 0.97)	0.89 (0.74, 0.99)	0.65
Dista phalanx—comminuted or fragmented fracture	External	10	0.66 (0.47, 0.82)	0.72 (0.58, 0.84)	0.69
	*p*-value (*)		0.65	0.65	
	Internal	49	0.75 (0.66, 0.84)	0.77 (0.69, 0.85)	0.73
Distal phalanx fracture	External	28	0.67 (0.58, 0.76)	0.68 (0.58, 0.79)	0.95
	*p*-value (*)		0.65	0.65	
	Internal	18	0.72 (0.57, 0.85)	0.69 (0.56, 0.82)	0.65
Joint subluxation	External	16	0.71 (0.53, 0.86)	0.70 (0.53, 0.88)	0.96
	*p*-value (*)		0.96	0.98	
	Internal	73	0.70 (0.63, 0.77)	0.71 (0.64, 0.78)	0.95
Fracture (all locations)	External	55	0.62 (0.54, 0.70)	0.65 (0.57, 0.73)	0.67
	*p*-value (*)		0.65	0.67	
	Internal	23	0.68 (0.55, 0.81)	0.73 (0.63, 0.81)	0.67
Swelling/dactylitis	External	47	0.57 (0.50, 0.66)	0.60 (0.51, 0.69)	0.71
	*p*-value (*)		0.65	0.65	
	Internal	32	0.64 (0.53, 0.73)	0.66 (0.56, 0.76)	0.65
Ossicles	External	36	0.42 (0.33, 0.53)	0.46 (0.36, 0.56)	0.67
	*p*-value (*)		0.17	0.18	
	Internal	16	0.61 (0.47, 0.74)	0.65 (0.53, 0.77)	0.71
Proximal phalanx fracture	External	23	0.46 (0.33, 0.58)	0.53 (0.41, 0.65)	0.65
	*p*-value (*)		0.65	0.65	
	Internal	11	0.59 (0.41, 0.75)	0.61 (0.39, 0.82)	0.95
Metacarpophalangeal joint subluxation	External	10	0.64 (0.44, 0.83)	0.61 (0.36, 0.85)	0.96
	*p*-value (*)		0.95	1	

Table organization as in Table 2

For the clavicle, the macro-averaged AUC was 0.80 (range, 0.59 to 0.95) for the inclusive and 0.81 (range, 0.63 to 0.94) for the exclusive model (*p *≥ 0.49). We observed high AUC values for common findings—labels with many positive cases—such as “Fracture (All Locations).” In contrast, less common findings had moderate AUC values, such as specific fracture locations like “Middle-Third Fracture,” and performance was poorer for rare findings like “Acromioclavicular Joint–Joint Space Widened.” Across labels, only minimal differences in AUC values were found between the inclusive and exclusive models, indicating that the handling of uncertain labels did not significantly affect model performance. The models maintained good performance on common findings in the external dataset, while challenging labels (e.g., “Swelling or Hematoma”) had weak performance on both internal and external datasets.

For the elbow, the macro-averaged AUC was 0.80 (range, 0.62 to 0.87) for the inclusive and 0.80 for the exclusive model (range, 0.61 to 0.88) (*p* ≥ 0.47). Again, common fracture-related findings (e.g., “Radial Head Fracture”) had high AUC values, while less prevalent findings, such as “Ossicles,” had lower and less consistent AUC values in the internal and external test sets. Model type and dataset did not significantly impact performance metrics.

For the thumb, the macro-averaged AUC was 0.76 (range, 0.59 to 0.91) for the inclusive and 0.78 for the exclusive model (range, 0.61 to 0.90) (*p* ≥ 0.65). AUC values were highest for joint degeneration-associated labels, both for the carpometacarpal and metacarpophalangeal articulations, while detecting fractures and soft tissue changes, such as “Swelling/Dactylitis,” was considerably less efficient, regardless of model type or dataset.

While no significant changes in AUC could be observed when comparing model type and dataset source, other metrics occasionally showed larger swings: For some, sensitivity increased while specificity decreased, or vice-versa (e.g., “Displacement” in the clavicle external set shows an 80% vs. 70% sensitivity shift that is offset by the opposite change in specificity). However, these fluctuations are tied to the single operating point imposed by Youden’s index. When the threshold-independent AUC was compared with the DeLong test, no label showed a significant inclusive–exclusive or internal–external difference.

## Discussion

This study used GPT-4o, a state-of-the-art LLM, for automatic label extraction from free-text radiologic reports of three anatomic regions of the upper extremity: radiography of the clavicle, elbow, and thumb. The LLM extracted labels with an accuracy ranging from 98.1% to 99.0%, which were used to train out-of-the-box multi-label classification models with competitive and robust diagnostic performance. However, detecting uncertain labels was less successful, with the LLM identifying only a portion of the uncertain cases identified manually, and label uncertainty did not significantly impact model performance.

Automatic label extraction allowed us to assemble training data rapidly and to build reliable classifiers for the frequent, bone-related labels: AUC values for “fracture,” “displacement,” and similar findings consistently exceeded 0.90. In contrast, performance on soft-tissue abnormalities (e.g., “swelling”,” soft tissue masses”) was markedly lower. These labels were both rarer and intrinsically harder to identify on radiographs. The limited number of positive cases, combined with greater subjectivity in the ground-truth reports, provided the network with weaker and noisier training data to learn from. As a result, confidence intervals widened, sensitivity and specificity fluctuated, and no statistically significant AUC differences emerged between the inclusive and exclusive uncertainty-handling schemes.

Our results confirm previous studies investigating automatic labeling approaches. One original archetypal approach is the CheXpert labeler, a rule-based NLP tool used for automatic label extraction of chest radiography reports and subsequent training of classification models [8]. While the CheXpert labeler and similar models (e.g., MIMIC-XCR labeler [23]) provide an efficient means to annotate large datasets, they are challenged by complex language, variable reporting styles, and negations and uncertainties, resulting in label inaccuracies of up to 10% [8, 10]. Recently, LLM-based methods for label extraction have been demonstrated to be more accurate due to their superior understanding of natural language [24]. Al Mohamed et al used an open-weight LLM to extract a single binary label, i.e., fracture present or not, from reports of ankle radiographs [13]. Their report-level accuracy of 92% was considerably higher than our study’s multi-label report-level accuracy of 74% to 86%. Their classification network trained on this label reached an AUC value of 0.93, comparable to our performance for some fracture labels. However, their study was limited to a single anatomic region and did not address label uncertainty.

Comparing our fracture-classification results to the meta-review in ref. [15], we note that the pooled AUC of 0.97 reported for classical fracture-detection CNNs, which is slightly above the 0.95 we achieved for fractures of the clavicle, was obtained using hand-curated datasets and models focusing on a single or very few labels. In contrast, our approach addressed a comprehensive multi-label prediction task for three upper-extremity regions (clavicle, elbow, and thumb), covering more than 25 distinct labels per region, including bone-related and soft tissue-related findings. These regions are imaged far less frequently than, for example, the chest. The principal added value of our study is therefore methodological: by combining LLM-based label extraction with routine radiologic reports, we demonstrate that competitive classifiers can be trained rapidly, even for sparsely imaged anatomic regions and a wide spectrum of clinically relevant findings, rather than just for fractures.

Handling uncertainty proved challenging. Although we aimed to standardize the uncertainty terms based on pertinent literature [14] and provided examples in the prompt, the LLM identified fewer uncertainty terms than were manually identified. Overall, the occurrence of uncertain labels in the ground truth was surprisingly low compared to other studies [14]. The interpretation of uncertainty is shaped by personal preference and experience, institutional policies, and healthcare system-related circumstances. For example, if the medicolegal environment is more litigious, as in the United States, uncertainty terms and hedging language may be more prevalent due to fear of malpractice litigation [25]. However, we found that uncertainty handling did not significantly impact model performance. In line with this observation, Irivin et al reported that converting uncertain labels to positive versus negative labels did not significantly impact diagnostic performance, except for atelectasis [8]. Because only a small fraction of labels were flagged as uncertain, the absence of performance differences between the inclusive and exclusive models may be due to limited statistical power instead of a true absence of effect. Nevertheless, the finding also suggests that our classifiers tolerate a modest level of label uncertainty without a measurable influence on performance.

This study has limitations. First, despite our intention to conduct a large-scale analysis, overall patient numbers were relatively low because of strict inclusion criteria. Data availability was insufficient for some labels, and we had to exclude those labels with fewer than 10 cases in the test sets. Even then, data scarcity for some labels led to wide confidence intervals and might have contributed to insignificant Delong test results. Second, we included German language reports; hence, whether the results hold when using less common languages is questionable. Fourth, the model did not provide visual outputs for model explainability. Fifth, model performance after training using automatically extracted labels was not compared to training using human reference labels, so the effect of LLM-based mislabeling remains unaccounted for. Sixth, the inherent non-deterministic nature of LLM outputs makes reproducing the results difficult. Seventh, although the terms “probable” and “less likely” differ in diagnostic certainty and confidence, the low prevalence of hedging language precluded reliable subdivision into multiple uncertainty levels. Therefore, future research should prioritize multi-institutional collaborations to assemble multi-label datasets for rare and subtle findings across more anatomic regions, conditions, modalities, and languages, and incorporate explainability tools such as class activation maps to enhance clinical transparency.

In conclusion, this study demonstrates that state-of-the-art LLMs can extract diagnostic labels from radiologic reports with high overall accuracy and use them to train competitive multi-label classification models for sparsely imaged anatomies such as the clavicle, elbow, and thumb. Unlike prior work limited to single labels or regions, our approach covers a broad spectrum of bone- and soft-tissue findings, incorporating an analysis of uncertainty handling that has shown minimal impact on performance. By enabling fully automated conversion of routine radiologic reports into structured, multi-label datasets, this workflow offers a scalable path for rapid model development and fine-tuning across institutions. With multi-institutional expansion, adaptation to additional modalities and languages, and integration of explainability tools, it has the potential to support clinically transparent and widely deployable AI in radiology.

## ELECTRONIC SUPPLEMENTARY MATERIAL

ELECTRONIC SUPPLEMENTARY MATERIAL

## Data Availability

The code for label extraction and model training is publicly available at https://github.com/TruhnLab/GPT_Label_Extraction_MSK.

## Abbreviations

CNN: Convolutional neural network
JSON: JavaScript object notation
LLMs: Large language models
NLP: Natural language processing
