# Supplementary material for: Large language model-based uncertainty-adjusted label extraction for artificial intelligence model development in upper extremity radiography
Source: Eur Radiol. 2025 Nov 14;36(4):2646–57. doi: 10.1007/s00330-025-12102-1 (PMC13035556; doi:10.1007/s00330-025-12102-1)

Large Language Model-Based Uncertainty-Adjusted Label Extraction for AI Model Development in Upper Extremity Radiography

ELECTRONIC SUPPLEMENTARY MATERIAL

Supplementary Text 1: Prompts for Automated Label Extraction

1) Clavicle:

Fill out the following template {template_json} in JSON format according to the information given in {finding}. Adhere strictly to the structure of the template. Only fill out the “finding” fields and do not add anything else.

1. Mark a finding as **true** only if it is explicitly confirmed in the report with no uncertainty or hedging terms.

2. Mark a finding as **uncertain** if the report contains hedging or uncertainty terms like “not clearly delineable” (German: “nicht sicher abgrenzbar”), “most likely” (“am ehesten”), “possibly” (“möglicherweise”), “cannot be ruled out” (“nicht ausgeschlossen”), “suspected” (“Verdacht auf”), “a potential differential diagnosis may be X” (“als DD käme in Frage X” or “DD X”) or similar phrases indicating doubt.

3. Mark a finding as **false** if the report explicitly states the absence of a finding or if the report contains no information related to the specific label.

4. If a specific sub-category finding (e.g., “Lateral Third Fracture”) is marked as true or uncertain, ensure that the broader category (e.g., “Fracture [All Locations]”) is marked accordingly.

5. Ensure no findings from other anatomic regions are considered when marking the “finding” fields. Each finding must be strictly limited to the anatomic region of interest (e.g., clavicle) specified in the template.

2) Elbow:

Fill out the following template {template_json} in JSON format according to the information given in {finding}. Adhere strictly to the structure of the template. Only fill out the “finding” fields and do not add anything else.

1. Mark a finding as **true** only if it is explicitly confirmed in the report with no uncertainty or hedging terms.

2. Mark a finding as **uncertain** if the report contains hedging or uncertainty terms like “not clearly delineable” (German: “nicht sicher abgrenzbar”), “most likely” (“am ehesten”), “possibly” (“möglicherweise”), “cannot be ruled out” (“nicht ausgeschlossen”), “suspected” (“Verdacht auf”), “a potential differential diagnosis may be X” (“als DD käme in Frage X” or “DD X”) or similar phrases indicating doubt.

3. Mark a finding as **false** if the report explicitly states the absence of a finding or if the report contains no information related to the specific label.

4. If a specific sub-category finding (e.g., “Radial Head Fracture”) is marked as true or uncertain, ensure that the broader category (e.g., “Fracture [All Locations]”) is marked accordingly.

5. Ensure no findings from other anatomic regions are considered when marking the “finding” fields. Each finding must be strictly limited to the anatomic region of interest (e.g., elbow) specified in the template.

3) Thumb

Fill out the following template {template_json} in JSON format according to the information given in {finding}. Adhere strictly to the structure of the template. Only fill out the “finding” fields and do not add anything else.

1. Mark a finding as **true** only if it is explicitly confirmed in the report with no uncertainty or hedging terms.

2. Mark a finding as **uncertain** if the report contains hedging or uncertainty terms like “not clearly delineable” (German: “nicht sicher abgrenzbar”), “most likely” (“am ehesten”), “possibly” (“möglicherweise”), “cannot be ruled out” (“nicht ausgeschlossen”), “suspected” (“Verdacht auf”), “a potential differential diagnosis may be X” (“als DD käme in Frage X” or “DD X”) or similar phrases indicating doubt.

3. Mark a finding as **false** if the report explicitly states the absence of a finding or if the report contains no information related to the specific label.

4. If a specific sub-category finding (e.g., “First Metacarpal Bone Fracture”) is marked as true or uncertain, ensure that the broader category (e.g., “Fracture [All Locations]”) is marked accordingly.

5. Ensure no findings from other anatomic regions are considered when marking the “finding” fields. Each finding must be strictly limited to the anatomic region of interest (e.g., thumb) specified in the template.

Supplementary Text 2: Templates for Automated Label Extraction for Radiography of the Clavicle, Elbow, and Thumb

For each label, the tool was instructed to choose from three options: “true,” i.e., the finding is present in the report; “false,” i.e., the finding is absent in the reports; or “uncertain,” i.e., the report contained phrases indicating doubt or hedging terms. “False” was the default option.

1) <u>Template for the Clavicle</u>


```
{
  “Fracture (All Locations)”: {
    “finding”: false
  },
  “Medial Third Fracture”: {
    “finding”: false
  },
  “Middle Third Fracture”: {
    “finding”: false
  },
  “Lateral Third Fracture”: {
    “finding”: false
  },
  “Comminuted or Fragmented Fracture (All Locations)”: {
    “finding”: false
  },
  “Displacement”: {
    “finding”: false
  },
  “Sclerotic Lesion”: {
    “finding”: false
  },
  “Lytic Lesion”: {
    “finding”: false
  },
  “Joint Dislocation (All Locations)”: {
    “finding”: false
  },
  “Joint Subluxation (All Locations)”: {
    “finding”: false
  },
  “Joint Degeneration (All Locations)”: {
    “finding”: false
  },
  “Acromioclavicular Joint - Joint Space widened”: {
    “finding”: false
  },
  “Acromioclavicular Joint - Joint Space narrowed”: {
    “finding”: false
  },
  “Acromioclavicular Joint - Subluxation”: {
    “finding”: false
  },
  “Acromioclavicular Joint - Dislocation”: {
    “finding”: false
  },
  “Acromioclavicular Joint Degeneration”: {
    “finding”: false
  },
  “Sternoclavicular Joint - Joint Space widened”: {
    “finding”: false
  },
  “Sternoclavicular Joint - Joint Space narrowed”: {
    “finding”: false
  },
  “Sternoclavicular Joint - Subluxation”: {
```

        “finding”: false
    },
    “Sternoclavicular Joint - Dislocation”: {
        “finding”: false
    },
    “Sternoclavicular Joint Degeneration”: {
        “finding”: false
    },
    “Swelling or Hematoma”: {
        “finding”: false
    },
    “Soft Tissue Calcifications”: {
        “finding”: false,
    },
    “Soft Tissues Masses or Mass-like lesions”: {
        “finding”: false
    },
    “Foreign Bodies”: {
        “finding”: false
    },
     “Ossicles”: {
        “finding”: false
    }
}

2) <u>Template for the Elbow</u>

{
    “Fracture (All Locations)”: {
        “finding”: false
    },
    “Lytic Lesion”: {
    “finding”: false
    },
    “Sclerotic Lesion”: {
        “finding”: false
    },
    “Distal Humerus - Fracture”: {
        “finding”: false
    },
    “Distal Humerus - Comminuted or Fragmented Fracture”: {
        “finding”: false
    },
    “Distal Humerus - Displacement”: {
        “finding”: false
    },
    “Distal Humerus Fracture - Extension into Joint”: {
        “finding”: false
    },
    “Olecranon Fracture”: {
        “finding”: false
    },
    “Olecranon - Displaced Fracture”: {
        “finding”: false
    },
    “Olecranon - Comminuted or Fragmented Fracture”: {
        “finding”: false
    },
    “Coronoid Process Fracture”: {
        “finding”: false
    },
    “Coronoid Process - Avulsion of the tip”: {
        “finding”: false

    },
    “Ulna Fracture”: {
        “finding”: false
    },
    “Radial Head Fracture”: {
        “finding”: false
    },
    “Radial Head - Displaced”: {
        “finding”: false
    },
    “Radial Head - Comminuted or Fragmented Fracture”: {
        “finding”: false
    },
    “Radial Neck Fracture”: {
        “finding”: false
    },
    “Radial Neck - Displaced”: {
        “finding”: false
    },
    “Radial Neck - Comminuted or Fragmented Fracture”: {
        “finding”: false
    },
    “Radius Fracture”: {
        “finding”: false
    },
    “Joint Subluxation (All Locations)”:”: {
        “finding”: false
    },
    “Joint Dislocation (All Locations)”:”: {
        “finding”: false
    },
    “Joint Degeneration (All Locations)”: {
        “finding”: false
    },
    “Soft Tissue Calcifications”: {
        “finding”: false
    },
    “Soft Tissue Masses or Mass-like lesions”: {
        “finding”: false
    },
    “Fat Pad Sign”: {
        “finding”: false
    },
    “Foreign Bodies”: {
        “finding”: false
    },
      “Ossicles”: {
        “finding”: false
    },
    “Exostosis”: {
        “finding”: false
    }
}

3) Template for the Thumb

{
    “Fracture (All Locations)”: {
        “finding”: false
    },
    “Comminuted or Fragmented Fracture (All Locations)”: {
        “finding”: false
    },
    “First Metacarpal Bone Fracture”: {
        “finding”: false

},
“First Metacarpal Bone - Comminuted or Fragmented Fracture”: {
“finding”: false
},
“Proximal Phalanx Fracture”: {
“finding”: false
},
“Proximal Phalanx - Comminuted or Fragmented Fracture”: {
“finding”: false
},
“Distal Phalanx Fracture”: {
“finding”: false
},
“Distal Phalanx - Comminuted or Fragmented Fracture”: {
“finding”: false
},
“Joint Subluxation (All Locations)”: {
“finding”: false
},
“Joint Dislocation (All Locations)”: {
“finding”: false
},
“Joint Degeneration (All Locations)”: {
“finding”: false
},
“Carpometacarpal Joint - Subluxation”: {
“finding”: false
},
“Carpometacarpal Joint - Dislocation”: {
“finding”: false
},
“Carpometacarpal Joint Degeneration”: {
“finding”: false
},
“Metacarpophalangeal Joint - Subluxation”: {
“finding”: false
},
“Metacarpophalangeal Joint - Dislocation”: {
“finding”: false
},
“Metacarpophalangeal Joint Degeneration”: {
“finding”: false
},
“Interphalangeal Joint - Subluxation”: {
“finding”: false
},
“Interphalangeal Joint - Dislocation”: {
“finding”: false
},
“Interphalangeal Joint Degeneration”: {
“finding”: false
},
“Swelling/Dactylitis”: {
“finding”: false
},
“Soft Tissue Calcifications”: {
“finding”: false
},
“Soft Tissues Masses or Mass-like lesions”: {
“finding”: false
},
“Foreign Bodies”: {
“finding”: false
},
“Ossicles”: {
“finding”: false
}

}

Supplementary Table S1: Training Details for the Classification Models.

Category	Description/Value
Framework	Pytorch 2.4.0
Pre-trained backbone	ResNet-50 (ImageNet-1k weights)
Input Size	512x512
Normalisation	mean [0.485 0.456 0.406], std [0.229 0.224 0.225]
Augmentations (train)	RandomHorizontalFlip (p = 0.5); RandomRotation ± 30°; ColorJitter
Optimizer	AdamW (lr = 1×10^{-4})
Learning Rate Schedule	StepLR (step_size 7, gamma = 0.1)
Loss	BCEWithLogitsLoss
Batch Size	32
Number of Epochs	30

Supplementary Table S2: Counts as a Function of Label, Dataset, and Split for Radiographs of the Clavicle.

Label	Inclusive Train	Exclusive Train	Inclusive Val	Exclusive Val	Internal Test	External Test
Fracture (All Locations)	379	379	95	95	121	147
Medial Third Fracture	27	22	7	6	7	14
Middle Third Fracture	211	207	52	51	66	76
Lateral Third Fracture	148	146	37	36	45	67
Comminuted or Fragmented Fracture (All Locations)	146	146	36	36	43	59
Displacement	331	329	83	83	98	123
Sclerotic Lesion	6	5	3	3	2	9
Joint Dislocation (All Locations)	15	15	4	4	9	16
Joint Subluxation (All Locations)	6	6	2	2	6	7
Joint Degeneration (All Locations)	48	48	12	12	19	33
Acromioclavicular Joint - Joint Space widened	39	38	10	10	14	34
Acromioclavicular Joint - Subluxation	18	18	4	4	6	5
Acromioclavicular Joint - Dislocation	32	30	7	7	8	16
Acromioclavicular Joint Degeneration	58	58	15	15	18	28
Swelling or Hematoma	31	30	7	7	12	34
Soft Tissue Calcifications	42	41	11	10	5	12
Foreign Bodies	16	16	5	5	7	13
Ossicles	8	7	2	2	2	4

Note: Train – training set, Val – validation set, Test – test set

Supplementary Table S3: Counts as a Function of Label, Dataset, and Split for Radiographs of the Elbow.

Label	Inclusive Train	Exclusive Train	Inclusive Val	Exclusive Val	Internal Test	External Test
Fracture (All Locations)	505	379	131	95	162	56
Lytic Lesion	8	8	3	2	3	2
Sclerotic Lesion	37	34	9	8	12	21
Distal Humerus Fracture	71	53	21	13	20	4
Distal Humerus - Comminuted or Fragmented Fracture	21	21	5	3	5	2
Distal Humerus - Displacement	29	28	9	8	10	3
Distal Humerus Fracture - Extension into Joint	13	12	5	3	3	1
Olecranon Fracture	95	82	30	21	25	6
Olecranon - Displaced Fracture	45	42	11	10	13	3
Olecranon - Comminuted or Fragmented Fracture	18	17	6	5	6	3
Coronoid Process Fracture	33	22	6	5	6	4
Coronoid Process - Avulsion of the tip	13	9	4	3	2	2
Ulna Fracture	59	52	18	13	30	9
Radial Head Fracture	388	312	97	78	102	42
Radial Head - Displaced	67	63	19	17	23	9
Radial Head - Comminuted or Fragmented Fracture	17	17	5	4	5	5
Radial Neck - Fracture	49	37	13	9	13	4
Radial Neck - Displaced	4	4	3	2	2	2
Radius Fracture	272	218	65	54	112	44
Joint Subluxation (All Locations)	10	8	4	3	2	1
Joint Dislocation (All Locations)	33	30	9	8	10	1
Joint Degeneration (All Locations)	215	210	56	53	52	39
Soft Tissue Calcifications	209	207	54	52	61	30
Soft Tissue Masses or Mass-like lesions	7	6	2	2	2	2
Fat Pad Sign	322	299	76	75	95	38
Foreign Bodies	50	50	12	12	20	8
Ossicles	75	71	18	18	19	16
Exostosis	73	72	19	18	57	40

Note: Train – training set, Val – validation set, Test – test set

Supplementary Table S4: Counts as a Function of Label, Dataset, and Split for Radiographs of the Thumb.

Label	Inclusive Train	Exclusive Train	Inclusive Val	Exclusive Val	Internal Test	External Test
Fracture (All Locations)	269	197	60	49	73	55
Comminuted or Fragmented Fracture (All Locations)	35	34	10	9	15	13
First Metacarpal Bone Fracture	37	22	7	5	8	7
Proximal Phalanx Fracture	75	50	15	12	16	23
Proximal Phalanx - Comminuted or Fragmented Fracture	7	6	5	5	2	3
Distal Phalanx Fracture	163	128	42	34	49	28
Distal Phalanx - Comminuted or Fragmented Fracture	28	27	8	7	14	10
Joint Subluxation (All Locations)	44	38	10	8	18	16
Joint Dislocation (All Locations)	19	19	4	4	7	4
Joint Degeneration (All Locations)	163	159	41	41	52	49
Carpometacarpal Joint - Subluxation	10	10	4	2	5	3
Carpometacarpal Joint Degeneration	101	101	25	25	30	30
Metacarpophalangeal Joint - Subluxation	24	18	5	5	11	10
Metacarpophalangeal Joint - Dislocation	6	6	1	1	2	1
Metacarpophalangeal Joint Degeneration	42	40	10	10	21	20
Interphalangeal Joint - Subluxation	22	18	4	4	3	2
Interphalangeal Joint - Dislocation	16	15	3	3	3	4
Interphalangeal Joint Degeneration	80	78	20	20	19	23
Swelling/Dactylitis	81	81	21	20	23	47
Soft Tissue Calcifications	37	34	8	8	7	15
Soft Tissues Masses or Mass-like lesions	5	5	1	1	1	0
Foreign Bodies	46	34	9	8	13	7
Ossicles	106	100	28	25	32	36

Note: Train – training set, Val – validation set, Test – test set

Supplementary Table S5: Counts of True, False, and Uncertain Labels in the Training and Validation Sets.

	True	False	Uncertain
Clavicle	1942	22378	42
Elbow	2958	83811	492
Thumb	1529	37865	231

Supplementary Table S6: Performance metrics of the inclusive and exclusive models for the clavicle for all labels with n≥10 in both datasets (internal dataset).

Label	N	AUC		Accuracy [%]		Sensitivity [%]		Specificity [%]	
		Inclusive	Exclusive	Inclusive	Exclusive	Inclusive	Exclusive	Inclusive	Exclusive
Fracture (All Locations)	121	0.95 (0.91, 0.97)	0.94 (0.90, 0.96)	87 (82, 91)	86 (82, 91)	80 (73, 87)	91 (85, 96)	94 (89, 98)	81 (74, 88)
Displacement	98	0.91 (0.87, 0.94)	0.90 (0.85, 0.94)	82 (77, 87)	85 (80, 89)	84 (76, 90)	88 (80, 94)	81 (75, 88)	83 (77, 89)
Middle Third Fracture	66	0.84 (0.78, 0.89)	0.86 (0.80, 0.91)	79 (73, 83)	80 (75, 85)	73 (62, 82)	65 (53, 76)	81 (75, 86)	86 (81, 91)
Lateral Third Fracture	45	0.83 (0.75, 0.89)	0.83 (0.76, 0.90)	79 (73, 85)	61 (55, 67)	69 (55, 82)	89 (80, 98)	81 (75, 87)	54 (48, 61)
Comminuted or Fragmented Fracture (All Locations)	43	0.89 (0.84, 0.93)	0.89 (0.84, 0.93)	74 (68, 79)	76 (71, 82)	95 (88, 100)	88 (79, 97)	69 (62, 76)	74 (67, 80)
Joint Degeneration (All Locations)	19	0.78 (0.65, 0.89)	0.76 (0.64, 0.87)	90 (86, 94)	82 (77, 87)	26 (8, 48)	47 (25, 71)	95 (92, 98)	85 (79, 89)
Acromioclavicular Joint Degeneration	18	0.78 (0.65, 0.89)	0.77 (0.64, 0.87)	81 (76, 86)	76 (71, 82)	56 (33, 78)	56 (31, 78)	83 (77, 88)	78 (73, 83)
Acromioclavicular Joint – Joint Space Widened	14	0.60 (0.46, 0.72)	0.63 (0.48, 0.76)	62 (56, 68)	70 (64, 76)	57 (31, 82)	50 (25, 78)	62 (56, 68)	71 (65, 77)
Swelling or Hematoma	12	0.59 (0.39, 0.75)	0.70 (0.55, 0.83)	45 (39, 51)	73 (68, 79)	75 (50, 100)	42 (13, 71)	43 (37, 50)	75 (69, 81)

Supplementary Table S7: Performance metrics of the inclusive and exclusive models for the clavicle for all labels with n≥10 in both datasets (external dataset).

Label	N	AUC		Accuracy [%]		Sensitivity [%]		Specificity [%]	
		Inclusive	Exclusive	Inclusive	Exclusive	Inclusive	Exclusive	Inclusive	Exclusive
Clavicle Fracture	147	0.91 (0.88, 0.94)	0.91 (0.88, 0.95)	84 (80, 88)	86 (82, 90)	73 (65, 80)	81 (74, 87)	95 (92, 98)	90 (85, 95)
Displacement	123	0.93 (0.90, 0.95)	0.92 (0.88, 0.95)	84 (80, 89)	83 (79, 87)	80 (72, 87)	70 (62, 77)	87 (82, 92)	93 (89, 96)
Middle Third Fracture	76	0.90 (0.86, 0.94)	0.88 (0.84, 0.92)	85 (81, 89)	81 (76, 85)	67 (56, 77)	50 (39, 61)	92 (88, 95)	91 (87, 95)
Lateral Third Fracture	67	0.81 (0.75, 0.87)	0.83 (0.77, 0.88)	71 (66, 76)	63 (58, 69)	73 (63, 84)	87 (78, 95)	71 (65, 76)	57 (51, 63)
Comminuted or Fragmented Fracture (All Locations)	59	0.91 (0.87, 0.94)	0.91 (0.87, 0.94)	79 (75, 84)	83 (79, 88)	92 (83, 98)	81 (71, 91)	76 (71, 81)	84 (79, 89)
Acromioclavicular Joint – Joint Space Widened	34	0.53 (0.42, 0.64)	0.51 (0.41, 0.62)	63 (57, 68)	69 (64, 74)	44 (27, 61)	26 (12, 42)	65 (60, 71)	74 (69, 80)
Swelling or Hematoma	34	0.47 (0.36, 0.58)	0.54 (0.42, 0.65)	60 (55, 66)	82 (77, 86)	41 (25, 58)	26 (11, 41)	63 (57, 69)	89 (85, 93)
Joint Degeneration (All Locations)	33	0.75 (0.68, 0.82)	0.76 (0.68, 0.83)	82 (77, 86)	77 (73, 82)	27 (13, 42)	42 (24, 59)	88 (84, 92)	82 (77, 86)
Acromioclavicular Joint – Signs of Degeneration	28	0.74 (0.65, 0.82)	0.74 (0.65, 0.82)	70 (65, 76)	74 (69, 79)	64 (46, 81)	57 (38, 77)	71 (65, 77)	75 (70, 81)

Supplementary Table S8: Performance metrics of the inclusive and exclusive models for the elbow for all labels with n≥10 in both datasets (internal dataset).

Label	N	AUC		Accuracy [%]		Sensitivity [%]		Specificity [%]	
		Inclusive	Exclusive	Inclusive	Exclusive	Inclusive	Exclusive	Inclusive	Exclusive
Fracture (All Locations)	162	0.87 (0.83, 0.90)	0.88 (0.85, 0.91)	80 (77, 83)	79 (76, 82)	75 (68, 82)	81 (75, 87)	81 (78, 84)	79 (75, 82)
Radius Fracture	112	0.86 (0.82, 0.90)	0.85 (0.82, 0.89)	79 (76, 82)	81 (78, 84)	76 (68, 83)	74 (66, 81)	80 (77, 83)	82 (79, 85)
Radial Head Fracture	102	0.86 (0.81, 0.90)	0.85 (0.81, 0.89)	84 (81, 86)	82 (80, 85)	69 (59, 78)	69 (60, 77)	86 (83, 89)	85 (82, 87)
Fat Pad Sign	95	0.78 (0.72, 0.84)	0.78 (0.72, 0.84)	78 (75, 81)	78 (75, 81)	62 (52, 72)	63 (54, 73)	80 (77, 83)	80 (77, 83)
Soft Tissue Calcifications	61	0.78 (0.71, 0.83)	0.76 (0.68, 0.83)	72 (69, 75)	67 (63, 70)	67 (55, 79)	75 (64, 86)	73 (69, 76)	66 (62, 69)
Exostosis	57	0.82 (0.76, 0.87)	0.81 (0.76, 0.86)	67 (63, 70)	61 (58, 65)	77 (66, 88)	84 (74, 93)	66 (62, 69)	60 (56, 63)
Joint Degeneration (All Locations)	52	0.81 (0.74, 0.86)	0.82 (0.75, 0.87)	79 (76, 82)	71 (68, 74)	69 (56, 81)	77 (65, 88)	80 (76, 82)	70 (67, 74)
Ossicles	19	0.62 (0.48, 0.74)	0.61 (0.48, 0.72)	54 (51, 58)	91 (89, 93)	63 (40, 83)	11 (0, 27)	54 (51, 58)	93 (91, 95)
Sclerotic Lesion	12	0.80 (0.66, 0.92)	0.85 (0.74, 0.94)	83 (80, 86)	70 (67, 73)	67 (38, 92)	83 (56, 100)	83 (80, 86)	70 (66, 73)

Supplementary Table S9: Performance metrics of the inclusive and exclusive models for the elbow for all labels with n≥10 in both datasets (external dataset).

Label	N	AUC		Accuracy [%]		Sensitivity [%]		Specificity [%]	
		Inclusive	Exclusive	Inclusive	Exclusive	Inclusive	Exclusive	Inclusive	Exclusive
Fracture (All Locations)	56	0.85 (0.79, 0.90)	0.85 (0.79, 0.90)	76 (71, 81)	76 (71, 81)	70 (57, 81)	86 (75, 94)	77 (72, 83)	74 (68, 79)
Radius Fracture	44	0.84 (0.76, 0.91)	0.88 (0.81, 0.94)	75 (70, 80)	80 (76, 84)	75 (63, 87)	82 (70, 93)	75 (70, 81)	80 (75, 84)
Radial Head Fracture	42	0.87 (0.80, 0.93)	0.89 (0.84, 0.94)	84 (80, 88)	85 (81, 89)	69 (55, 83)	83 (71, 93)	87 (83, 91)	85 (81, 89)
Exostosis	40	0.86 (0.80, 0.91)	0.85 (0.78, 0.91)	73 (68, 78)	58 (52, 63)	95 (88, 100)	93 (84, 100)	70 (65, 75)	52 (46, 58)
Fat Pad Sign	38	0.78 (0.68, 0.86)	0.78 (0.69, 0.86)	68 (62, 73)	69 (64, 74)	71 (56, 85)	71 (55, 85)	67 (61, 73)	69 (63, 74)
Joint Degeneration (All Locations)	39	0.84 (0.77, 0.92)	0.84 (0.77, 0.91)	81 (76, 85)	71 (66, 77)	74 (60, 88)	82 (69, 93)	82 (77, 87)	70 (64, 75)
Soft Tissue Calcifications	30	0.61 (0.49, 0.71)	0.63 (0.52, 0.73)	75 (70, 80)	68 (63, 74)	37 (19, 53)	53 (36, 71)	80 (74, 84)	70 (65, 75)
Sclerotic Lesion	21	0.65 (0.53, 0.77)	0.63 (0.52, 0.75)	72 (66, 77)	60 (55, 65)	43 (19, 67)	52 (32, 75)	74 (69, 79)	61 (55, 66)
Ossicles	16	0.78 (0.66, 0.90)	0.75 (0.60, 0.87)	60 (55, 66)	92 (89, 95)	75 (53, 94)	19 (0, 38)	60 (54, 65)	96 (94, 99)

Supplementary Table S10: Performance metrics of the inclusive and exclusive models for the thumb for all labels with n≥10 in both datasets (internal dataset).

Label	N	AUC		Accuracy [%]		Sensitivity [%]		Specificity [%]	
		Inclusive	Exclusive	Inclusive	Exclusive	Inclusive	Exclusive	Inclusive	Exclusive
Fracture (All Locations)	73	0.70 (0.63, 0.77)	0.71 (0.64, 0.78)	77 (73, 81)	63 (58, 68)	51 (39, 62)	66 (55, 77)	83 (79, 87)	63 (57, 68)
Joint Degeneration (All Locations)	52	0.89 (0.84, 0.93)	0.88 (0.81, 0.93)	83 (80, 87)	80 (76, 84)	85 (74, 93)	83 (71, 92)	83 (79, 87)	79 (75, 84)
Distal Phalanx Fracture	49	0.75 (0.66, 0.84)	0.77 (0.69, 0.85)	80 (76, 84)	77 (73, 81)	49 (34, 62)	65 (52, 78)	84 (80, 88)	78 (74, 83)
Ossicles	32	0.64 (0.53, 0.73)	0.66 (0.56, 0.76)	56 (51, 60)	60 (55, 65)	59 (41, 76)	63 (44, 80)	55 (50, 60)	60 (55, 65)
Carpometacarpal Joint Degeneration	30	0.91 (0.85, 0.96)	0.90 (0.85, 0.95)	81 (77, 85)	81 (77, 85)	87 (74, 97)	87 (74, 97)	81 (77, 85)	81 (77, 85)
Swelling/Dactylitis	23	0.68 (0.55, 0.81)	0.73 (0.63, 0.81)	89 (86, 92)	75 (71, 79)	26 (10, 44)	52 (30, 73)	93 (91, 96)	76 (72, 80)
Metacarpophalangeal Joint Degeneration	21	0.88 (0.78, 0.95)	0.89 (0.79, 0.96)	80 (77, 84)	92 (89, 94)	81 (63, 96)	67 (44, 86)	80 (76, 84)	93 (90, 95)
Interphalangeal Joint Degeneration	19	0.85 (0.76, 0.93)	0.84 (0.73, 0.92)	75 (71, 79)	79 (75, 83)	84 (65, 100)	74 (53, 93)	75 (71, 79)	79 (75, 83)
Joint Subluxation	18	0.72 (0.57, 0.85)	0.69 (0.56, 0.82)	92 (89, 94)	92 (89, 94)	11 (0, 28)	11 (0, 27)	96 (93, 98)	96 (94, 98)
Proximal Phalanx Fracture	16	0.61 (0.47, 0.74)	0.65 (0.53, 0.77)	35 (31, 40)	91 (88, 93)	81 (58, 100)	13 (0, 31)	33 (28, 38)	94 (92, 96)
Comminuted or Fragmented Fracture (All Locations)	15	0.80 (0.63, 0.94)	0.86 (0.71, 0.98)	87 (83, 90)	76 (72, 80)	60 (36, 85)	80 (56, 100)	88 (85, 91)	76 (72, 80)
Distal Phalanx - Comminuted or Fragmented Fracture	14	0.85 (0.69, 0.97)	0.89 (0.74, 0.99)	88 (85, 92)	91 (88, 94)	71 (45, 100)	86 (67, 100)	89 (86, 92)	91 (88, 94)
Metacarpophalangeal Joint Subluxation	11	0.59 (0.41, 0.75)	0.61 (0.39, 0.82)	65 (60, 69)	87 (84, 90)	55 (25, 86)	27 (0, 56)	65 (60, 69)	89 (86, 92)

Supplementary Table S11: Performance metrics of the inclusive and exclusive models for the thumb for all labels with n≥10 in both datasets (external dataset).

Label	N	AUC		Accuracy [%]		Sensitivity [%]		Specificity [%]	
		Inclusive	Exclusive	Inclusive	Exclusive	Inclusive	Exclusive	Inclusive	Exclusive
Fracture (All Locations)	55	0.62 (0.54, 0.70)	0.65 (0.57, 0.73)	75 (69, 79)	69 (64, 74)	27 (16, 38)	45 (33, 58)	85 (81, 89)	75 (69, 80)
Joint Degeneration (All Locations)	49	0.90 (0.85, 0.95)	0.91 (0.86, 0.95)	83 (79, 87)	71 (65, 76)	90 (81, 98)	92 (83, 98)	82 (77, 87)	67 (60, 73)
Swelling/Dactylitis	47	0.57 (0.50, 0.66)	0.60 (0.51, 0.69)	78 (73, 82)	62 (57, 68)	6 (0, 15)	53 (39, 68)	91 (87, 94)	64 (58, 70)
Ossicles	36	0.42 (0.33, 0.53)	0.46 (0.36, 0.56)	31 (26, 36)	38 (33, 43)	58 (41, 73)	56 (39, 73)	27 (22, 32)	36 (30, 41)
Carpometacarpal Joint Degeneration	30	0.90 (0.84, 0.95)	0.90 (0.85, 0.95)	81 (77, 85)	76 (71, 80)	77 (61, 91)	93 (83, 100)	81 (77, 86)	74 (68, 79)
Distal Phalanx Fracture	28	0.67 (0.58, 0.76)	0.68 (0.58, 0.79)	84 (80, 88)	80 (75, 84)	14 (3, 29)	39 (23, 60)	91 (87, 94)	84 (79, 88)
Interphalangeal Joint Degeneration	23	0.89 (0.82, 0.94)	0.89 (0.83, 0.94)	77 (73, 82)	77 (73, 81)	91 (78, 100)	91 (78, 100)	76 (71, 81)	76 (71, 81)
Proximal Phalanx Fracture	23	0.46 (0.33, 0.58)	0.53 (0.41, 0.65)	36 (31, 42)	91 (87, 94)	65 (45, 84)	0 (0, 0)	34 (28, 40)	98 (96, 100)
Metacarpophalangeal Joint Degeneration	20	0.85 (0.75, 0.92)	0.85 (0.77, 0.93)	84 (79, 88)	89 (86, 92)	80 (62, 95)	40 (17, 63)	84 (80, 88)	93 (89, 95)
Joint Subluxation	16	0.71 (0.53, 0.86)	0.70 (0.53, 0.88)	94 (91, 96)	93 (90, 96)	13 (0, 33)	13 (0, 33)	98 (97, 100)	98 (96, 99)
Comminuted or Fragmented Fracture (All Locations)	13	0.69 (0.56, 0.81)	0.73 (0.64, 0.81)	85 (81, 89)	73 (67, 77)	15 (0, 37)	46 (18, 75)	89 (84, 92)	74 (69, 78)
Distal Phalanx - Comminuted or Fragmented Fracture	10	0.66 (0.47, 0.82)	0.72 (0.58, 0.84)	86 (82, 90)	89 (85, 92)	10 (0, 33)	10 (0, 36)	88 (84, 92)	92 (89, 95)
Metacarpophalangeal Joint Subluxation	10	0.64 (0.44, 0.83)	0.61 (0.36, 0.85)	71 (66, 76)	93 (90, 96)	50 (20, 83)	30 (0, 64)	72 (67, 77)	95 (92, 97)

Supplementary Figure S1: Receiver-operating characteristic -and precision recall-curves of the Inclusive and Exclusive Models for the Clavicle for all Labels with n≥10 in Both Datasets.

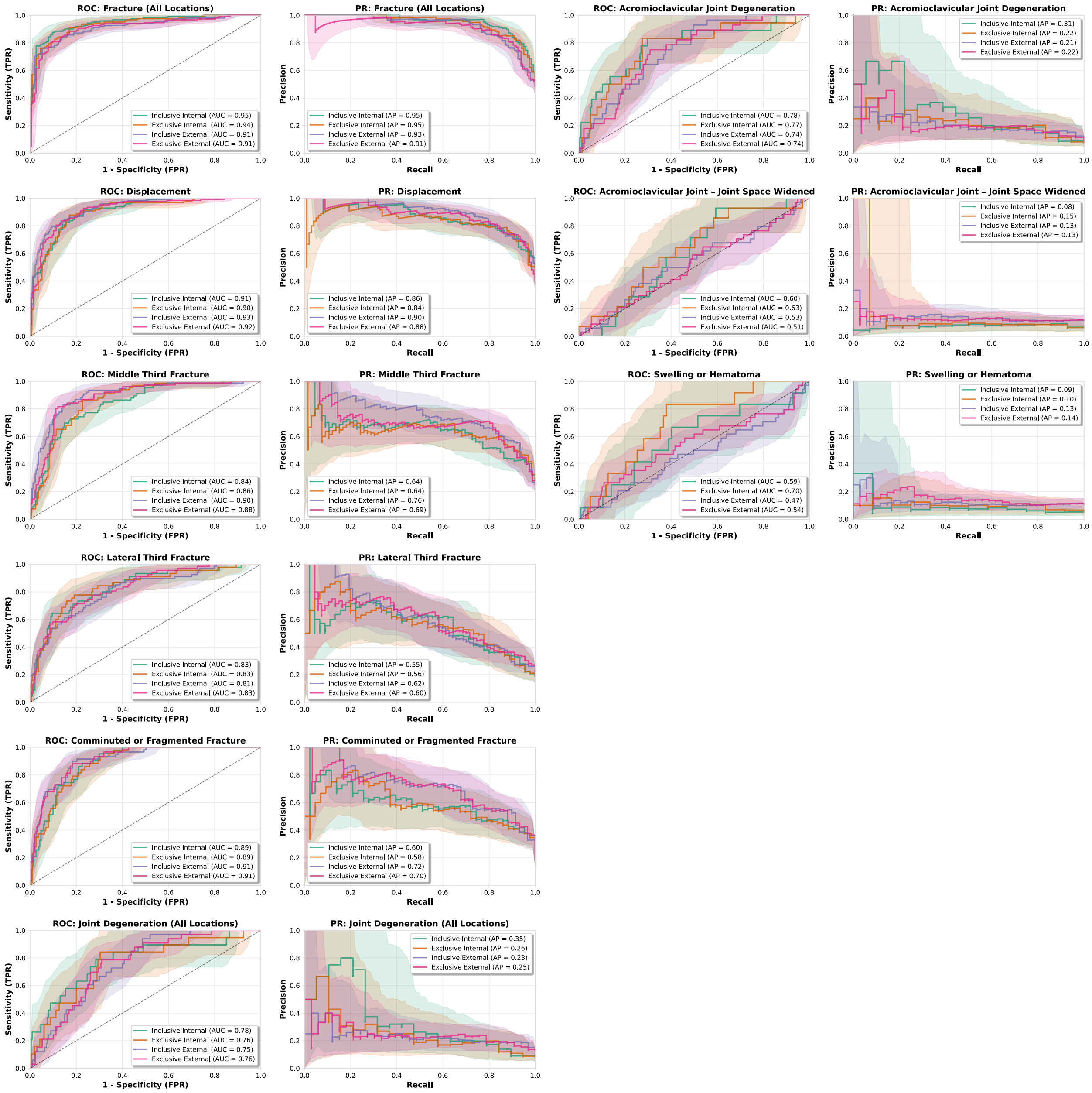

Supplementary Figure S2: Receiver-operating characteristic -and precision recall-curves of the inclusive and exclusive models for the elbow for all labels with n≥10 in both datasets.

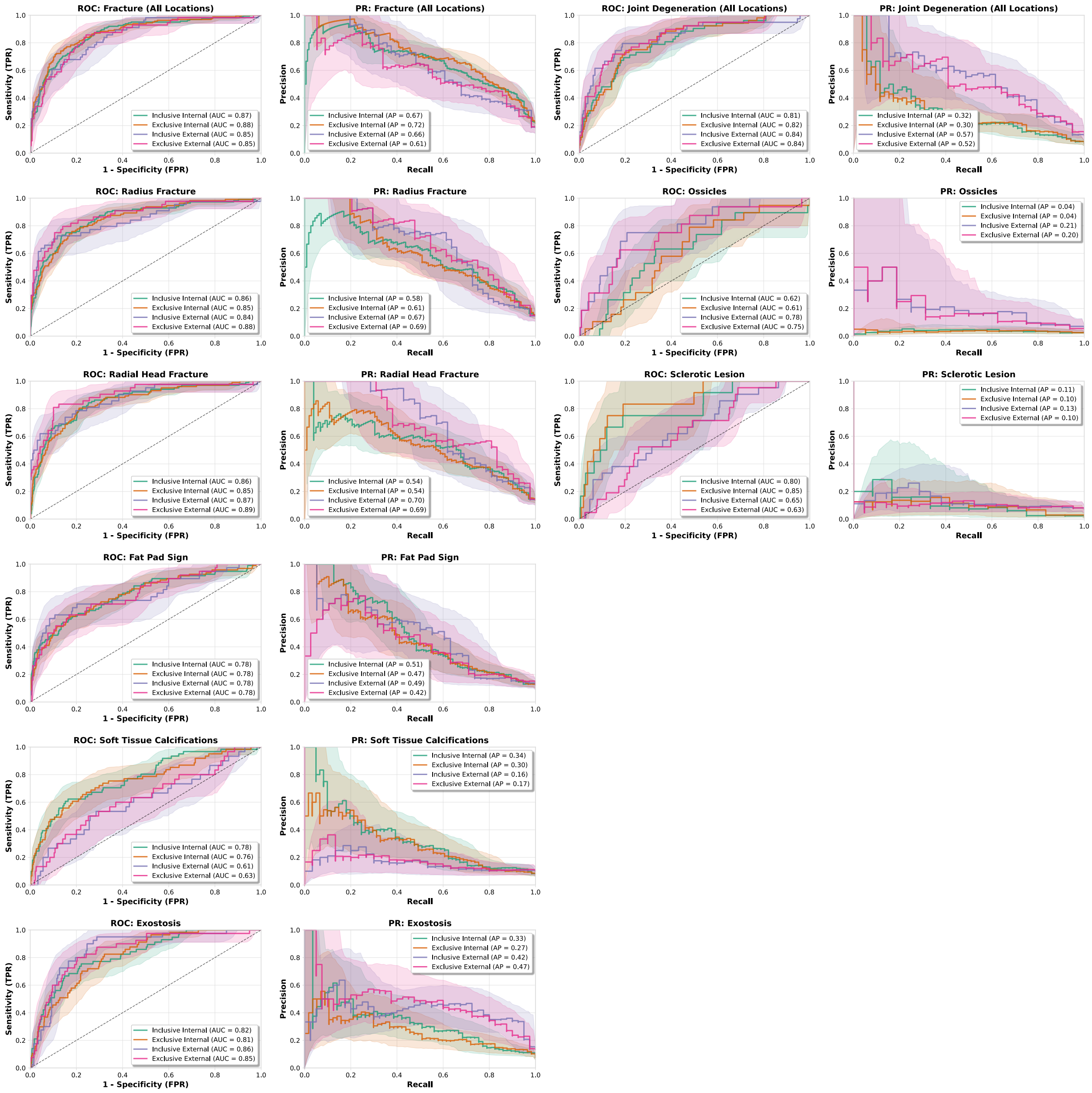

Supplementary Figure S3: Receiver-operating characteristic -and precision recall-curves of the inclusive and exclusive models for the thumb for all labels with n≥10 in both datasets.

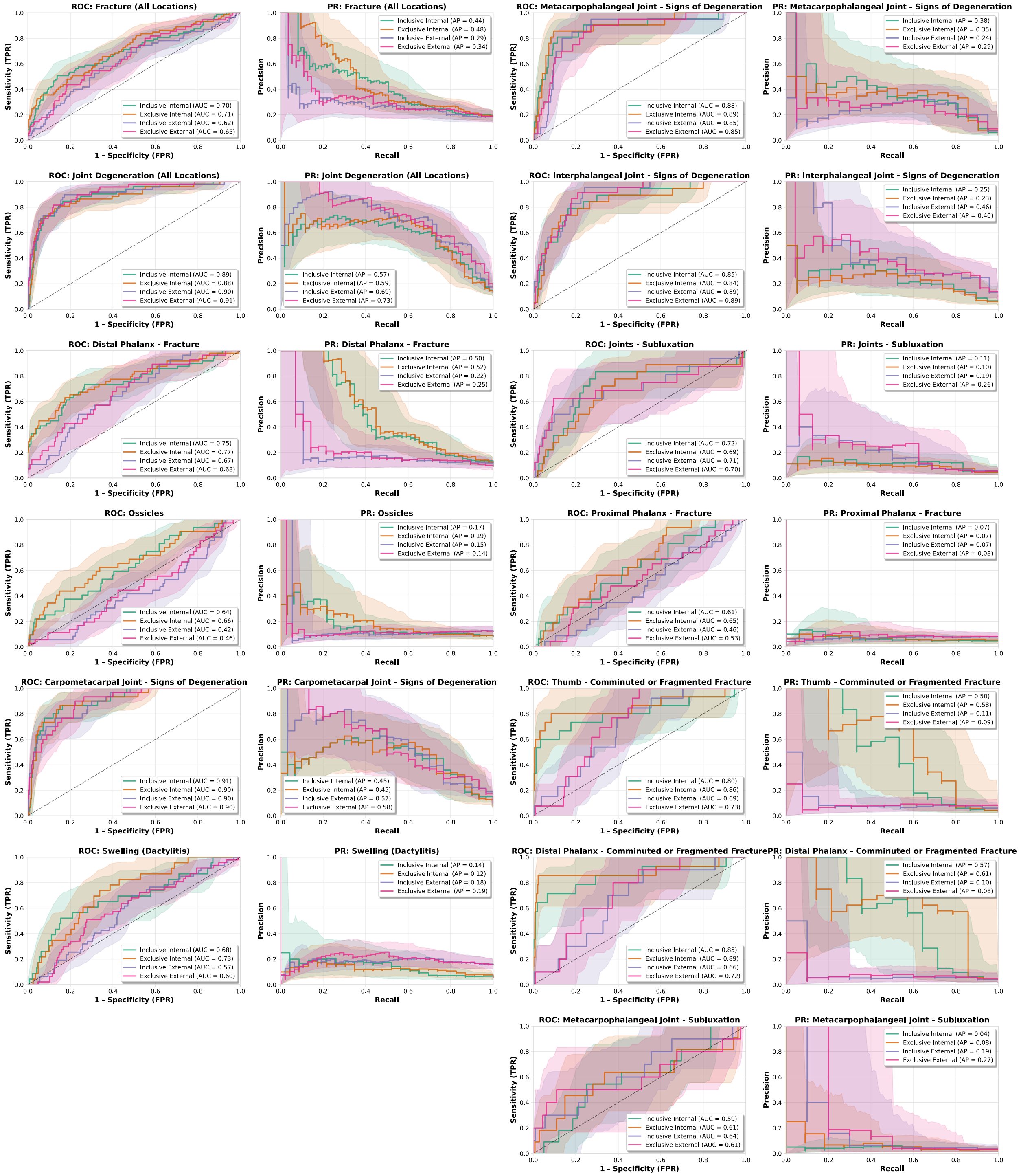

Supplement: Supplementary file 1 — ELECTRONIC SUPPLEMENTARY MATERIAL [file 330_2025_12102_MOESM1_ESM.pdf]
